# Zika Virus May Affect the Universal Two-Child Policy: A New Challenge for China

**DOI:** 10.1371/journal.pntd.0004984

**Published:** 2016-11-10

**Authors:** Pengcheng Zhou, Juan Wang, Yixiang Zheng, Rongrong Zhou, Xue-Gong Fan

**Affiliations:** Department of Infectious diseases, Key Laboratory of Viral Hepatitis of Hunan province, Xiangya Hospital, Central South University, Changsha, Hunan, P.R. China; The Hospital for Sick Children, CANADA

Toward the end of 2015, China relaxed its more than three-decade-old one-child policy to address the problems of a rapidly aging population, now allowing all couples to have two children (the universal two-child policy) [1]. It is expected that China will witness a huge baby boom in the next few years [2]. However, the potential threat of Zika virus may affect the anticipated baby boom.

As of August 3, 2016, more than 1.5 million people in 68 countries and territories have been affected by Zika virus, including 51 countries and territories with a first reported outbreak since2015 [3]. Among them, the United States reported mosquito-borne Zika virus transmission for the first time on July 29, 2016. Fourteen countries or territories have reported microcephaly and other central nervous system (CNS) malformations to be potentially associated with Zika virus infection or suggestive of congenital infection [3,4].

The primary transmission vectors of Zika virus are *Aedes aegypti* and *A*. *albopictus*; however, mounting evidence shows that the virus may be transmitted through *Culexpipiens* and *A*. *triseriatus*, sexual intercourse, blood transfusion, laboratory exposure, and from mother to child [3,5,6]. On February 1, 2016, the World Health Organization declared Zika virus to be a Public Health Emergency of International Concern [4], and many technical guidances, including case definition for Zika virus, information for travelers visiting Zika-affected countries, pregnancy management in the context of Zika virus, and prevention of sexual transmission of Zika virus have been released [7].

**Fig 1:**
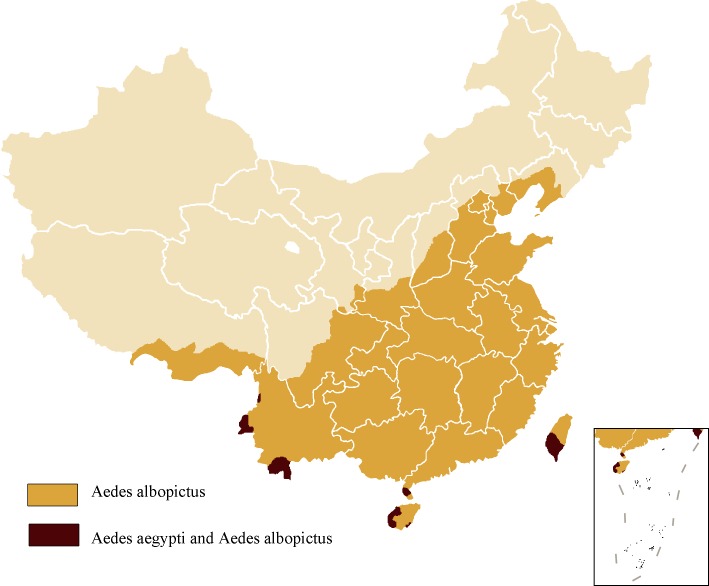
Geographical distribution map of *A*. *albopictus* and *A*. *aegypti* in China. *A*. *albopictus* could be found in all the provinces of China, except Xinjiang, Inner Mongolia, Ningxia, Qinghai, Heilongjiang, and Jilin, and *A*. *aegypti* is only distributed in Yunnan, Guangdong, Hainan, and Taiwan.

Both Zika and dengue viruses are primarily transmitted by the *Aedes* mosquitoes; therefore, Zika virus may attain the same geographical spread as dengue virus [8]. Considering the fact that cases of dengue have been reported in China during each of the past 25 years and have reached a historical high in 2014 (47,056 cases) [9], it is possible for a Zika virus epidemic to occur in China. In particular, the following contributing factors are worth noting. First, China has the primary transmission vectors (*A*. *aegypti* and *A*. *albopictus*) (Fig 1) and a suitable climate for the prevalence of Zika virus. Besides, most of the Chinese people have not acquired immunity against the virus. Second, mosquitoes or larvae infected with Zika virus may be introduced to China with commodities [10].China is the largest trading nation in the world. It has been reported that some of its major trading partners have cases of Zika virus infection, including Brazil (the most affected country). Third, Zika virus can also spread to China through asymptomatic cases because it is impossible to check all inbound travelers. In fact, 22 imported cases were reported in mainland China between February 9, 2016, and July 12, 2016 [11,12]. The rapid development of business and tourism led to an increase in travel, with 523 million people exiting and entering mainland China in 2015, including 51.92 million foreigners [13]. In addition, many Chinese people will travel to Brazil for the 2016 Summer Olympics in Rio de Janeiro [14].Fourth, adult mosquitoes carrying Zika virus may enter China from the neighboring countries because there has been evidence of Zika virus transmission in Laos, Vietnam, and Thailand. Lastly, China may also have local transmission of Zika virus, considering that no large-scale epidemiological survey has been conducted in China.

An outbreak of Zika virus is most likely to occur in the southern and southwestern parts of China (the dengue fever epidemic areas, such as the Guangdong and Hainan provinces) and in large cities with a large inbound population during the mosquito season. These regions have a very high population density, with tens of millions of women in their reproductive age. Therefore, if a Zika virus outbreak coincides with the baby boom, serious consequences, including increases in birth defects and delayed pregnancy, may occur. This may lead to a baby boom that is smaller than anticipated and a heavy societal burden in the long run, contrary to the original intention of the universal two-child policy.

A Zika virus outbreak in China is possible, and public health preparedness is urgently needed. The government could apply lessons learned from controlling dengue to prevent an outbreak of Zika virus. Proactive vector control measures should be taken, and medical supplies should be prepared. Inspection and quarantine policies at entry ports should be strengthened. Moreover, Chinese scientists should participate in international efforts to accelerate vaccine development. A large-scale epidemiology survey should be conducted to identify potential local cases of Zika virus infection. Mass media should be used to educate the public about Zika virus disease prevention, including protection against mosquito bites and safe sex as the main measures to avoid infection.

Fortunately, the Chinese government is aware of this situation, and many measures have been implemented in response to the possible challenges posed by Zika virus. A national scheme on the prevention and control of Zika virus disease and a national scheme on the diagnosis and treatment of Zika virus disease has been worked out in China in February 2016 [15, 16]. Also, an announcement pertaining to the prevention of Zika virus diseases from spreading to China has been released by the eight ministries and commissions of China on March 2, 2016 [17], and inspection and quarantine at entry ports has been strengthened since then. Besides, all the imported cases have been identified, isolated, and treated timely and accurately, and all the patients have recovered and have been released from hospitals.
